# Early predictors of cognitive trajectories from birth to adolescence in children born extremely preterm

**DOI:** 10.1111/dmcn.70211

**Published:** 2026-02-26

**Authors:** Stephen R. Hooper, Robert M. Joseph, T. Michael O′Shea, Karl C. K. Kuban, Rachana Singh, Jean A. Frazier, Genevieve Taylor, Xianming Tan, Rebecca C. Fry, Hudson P. Santos, the ELGAN Study Investigators

**Affiliations:** ^1^ Departments of Health Sciences and Psychiatry, School of Medicine University of North Carolina‐Chapel Hill Chapel Hill NC USA; ^2^ Department of Anatomy and Neurobiology Boston University Chobanian and Avedisian School of Medicine Boston MA USA; ^3^ Department of Pediatrics University of North Carolina‐Chapel Hill Chapel Hill NC USA; ^4^ Department of Pediatrics Boston Medical Center Boston MA USA; ^5^ Department of Pediatrics Tufts University School of Medicine Boston MA USA; ^6^ Eunice Kennedy Shriver Center University of Massachusetts Chan Medical School Worcester MA USA; ^7^ Department of Biostatistics University of North Carolina‐Chapel Hill Chapel Hill NC USA; ^8^ Department of Environmental Sciences and Engineering, Gillings School of Global Public Health University of North Carolina‐Chapel Hill Chapel Hill NC USA; ^9^ School of Nursing and Health Studies University of Miami Coral Gables FL USA

## Abstract

**Aim:**

To predict trajectories of cognitive abilities from ages 2 years to 15 years in children born extremely preterm.

**Method:**

This longitudinal cohort study examined 659 (330 males) children born at 23 weeks to 27 weeks' gestation with cognitive assessments at ages 2 years, 10 years, and 15 years. Early factors analysed included maternal and neonatal characteristics, comorbidities, and neonatal inflammatory proteins.

**Results:**

For verbal, non‐verbal, and combined cognitive abilities, greater social disadvantage at birth, male sex, 23 weeks to 24 weeks gestational age, bronchopulmonary dysplasia (BPD), ultrasound‐detected cerebral white matter injury (WMI), and elevated inflammatory proteins in neonatal blood were associated with lower scores over the 15‐year time frame for all outcomes. Analyses of joint association between socially disadvantaged groups and WMI suggested that the effect of no social disadvantage was evident only among children without WMI, whereas the effect of WMI was evident irrespective of social disadvantage.

**Interpretation:**

In this cohort of children born extremely preterm, social disadvantage at birth, male sex, BPD, WMI, and neonatal systemic inflammation were associated with slower gains in cognitive scores from age 2 years to 15 years. Early WMI moderated the associations between social disadvantage and cognitive functioning. Efforts to address both neonatal morbidities and social factors could improve cognitive functioning in children born extremely preterm.

## Graphical abstract

At 2 years, 10 years, and 15 years, the presence of early white matter injury (WMI) moderated associations between lower social risk and cognitive functioning in the social disadvantage index group x WMI interactions for combined abilities. Findings suggest that the benefits of low social risk were present in those without early WMI, but are potentially attenuated when early WMI was detected; nonetheless, positive cognitive growth continued to be present for the early WMI group across the three time points.

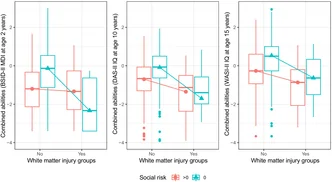

**This original article is commented by Wolke on pages** 1493–1494 **of this issue.**

**Abbreviations**
BPD: bronchopulmonary dysplasia
BSID‐II: Bayley Scales of Infant Development, Second Edition
ELGAN: Extremely Low Gestational Age Newborn
MDI: Mental Development Index
WMI: white matter injury

**What this paper adds**

- Social disadvantage and medical factors predicted less gain in cognitive abilities.
- Lower functioning at age 2 years predicted slower gain in verbal abilities.
- Early white matter injury attenuated the associations between no social disadvantage and cognitive gains.
- Bronchopulmonary dysplasia and neonatal systemic inflammation predicted decreased cognitive gains over time.

Neonates born extremely preterm (i.e. those born before less than 28 weeks' gestation) frequently experience severe neonatal challenges, such as bronchopulmonary dysplasia (BPD), bacteremia, and cerebral white matter injury (WMI), which increase the risk of cognitive impairment.^1^, ^2^, ^3^, ^4^, ^5^, ^6^, ^7^, ^8^ Longitudinal studies of cognitive trajectories in children born extremely preterm through adolescence found that their cognitive abilities do not improve substantially from infancy into young adulthood.^9^ Furthermore, neonates born extremely preterm are more likely than peers born at term to experience socioeconomic disadvantages that, in turn, can affect cognitive and behavioral outcomes.^10^, ^11^, ^12^, ^13^, ^14^, ^15^, ^16^, ^17^, ^18^

While exposure to beneficial environmental factors has a positive impact on cognitive functioning, particularly in the preschool years,^9^ it is unclear how early neonatal complications (e.g. BPD, bacteremia, WMI) might interact with social disadvantage factors (e.g. food insecurity) to affect cognitive outcomes. To date, few studies have examined the relationship between early social disadvantage factors and neonatal comorbidities with respect to moderation of cognitive outcomes, especially with increasing age into the adolescent years. Based on earlier findings,^10^, ^12^ it may be expected that known associations with neonatal morbidity might be attenuated among children growing up in households with more socioeconomic resources.

This article addresses this knowledge gap using data from the Extremely Low Gestational Age Newborn (ELGAN) study. The ELGAN study is the largest longitudinal cohort study tracking infants born extremely preterm in the USA into middle adolescence, with measures of development and cognitive function at ages 2 years, 10 years, and 15 years. We hypothesized that verbal, non‐verbal, and overall cognitive scores would be stable over the period from 2 years to 15 years (i.e. age‐based standard scores transformed to *z*‐scores), and that social disadvantage, neonatal medical conditions (e.g. WMI), and neonatal systemic inflammation would be associated with slower gains in cognitive scores over time. In addition, given the known impact of social disadvantage factors on the development of cognitive abilities in children born extremely preterm,^9^ and on the brain development of populations born preterm more generally,^19^, ^20^, ^21^, ^22^ we examined whether social disadvantage would interact with three of the more prevalent neonatal medical disorders (i.e. BPD, bacteremia, WMI) with respect to their impact on cognitive trajectories through middle adolescence.

## METHOD

### Participants

The ELGAN study is a longitudinal cohort study that enrolled participants based on a gestational age of 23 weeks to 27 weeks. A total of 1506 infants were enrolled in 2002 to 2004 from 14 hospitals in five states in the USA (Figure S1). Of the 1198 children who survived to 2 years of age, 802 returned for both the 2‐year and the 10‐year follow‐ups and participated in at least one of the cognitive assessments. For the age 15 years visits, 143 were lost to follow‐up or not tested at that time, leaving 659 having at least two cognitive measures across the three time points, comprising over 1700 visits. Approval for participation was obtained using written parental consent and, when applicable, written child assent. All aspects of the study were approved by the institutional review board at each site.

### Measures

The measures for this study represented age‐appropriate, standardized measures of developmental and cognitive functioning. For each measure, age‐based standard scores from the test normative data were derived and transformed into *z*‐score equivalents. All measures were collected by trained clinical examiners at each site.

The Bayley Scales of Infant Development, Second Edition (BSID‐II) was administered at approximately 2 years (adjusted for the degree of preterm birth). While the BSID‐II provides both the Mental Development Index (MDI) and the Psychomotor Development Index, we elected to use the MDI as the measure at age 2 years. For the longitudinal design, given the array of verbal and non‐verbal test items at age 2 years, the MDI served as a downward proxy for verbal, non‐verbal, and combined abilities in the analyses. The BSID‐II MDI maintains a strong correlation (*r* = 0.61) with later cognitive functioning.^23^

The Verbal and Nonverbal Reasoning subscales of the Differential Ability Scales, Second Edition were administered at age 10 years and were averaged to estimate general cognitive ability. The Differential Ability Scales, Second Edition are strongly correlated (*r* = 0.84) with the Wechsler Abbreviated Scales of Intelligence, Second Edition.

The Wechsler Abbreviated Scales of Intelligence, Second Edition was administered at age 15 years. The two‐subtest form consisted of Vocabulary (verbal abilities) and Matrix Reasoning (non‐verbal abilities), and provided an estimate of the Full‐scale IQ (i.e. combined cognitive abilities).

### Early‐life factors

Early‐life predictors of cognitive outcomes were selected based on published research:^10^, ^11^, ^12^, ^13^, ^14^, ^15^ maternal sociodemographic characteristics; newborn child characteristics; and neonatal comorbidities. Maternal characteristics were ascertained via maternal interview within a few days of delivery of the ELGAN participant and included a social disadvantage index that included (1) less than 12 years of education, (2) public health insurance (e.g. Medicaid), (3) marital status (single), and (4) receipt of supplemental nutrition assistance (e.g. food stamps). This social disadvantage index ranged from 0 to 4, with higher scores reflecting higher social risk.^24^ The self‐reported ethnicity of the mother was collected for sample description. Sex, gestational age, birthweight *z*‐score, and multiple births were used as covariates in the models. Also included was an index of neonatal systemic inflammation that we have used in prior studies involving the ELGAN cohort. This was calculated using the number of repeated elevations of inflammation‐related proteins, defined as being in the highest quartile on at least two of three measurements made on postnatal days 1, 7, and 14.^25^, ^26^ For this inflammation index, six proteins were considered, so the range of values is 0 to 6: C‐reactive protein, serum amyloid, interleukin‐6, interleukin‐8, tumor necrosis factor, and intercellular adhesion molecule‐1. Elevations of these proteins have been associated with cognitive impairment at ages 2 years and 10 years in the ELGAN cohort^25^, ^26^ and in other samples of children born extremely preterm.^27^, ^28^ Finally, bacteremia, BPD, and WMI were included in the model given their prior identified importance to later cognitive functioning in children born extremely preterm.^7^, ^11^, ^12^, ^13^ Bacteremia (classified as none, suspected, or definite) and BPD (classified as mild to moderate if supplemental oxygen was required at 36 weeks postmenstrual age and as severe if mechanical ventilation was required)^12^ were identified via medical records, while cranial ultrasound‐identified WMI was defined as echolucent lesions of cerebral white matter or ventricular enlargement persisting after the second postnatal week.^29^

### Statistical analyses

Descriptive statistics, means and SDs for numerical variables, and frequencies for categorical variables, were generated for all maternal and newborn child characteristics.

For the verbal, non‐verbal, and combined cognitive scores, change over time and its associations with early‐life factors were examined using linear mixed effect models with a random participant effect for each newborn child and a random family effect for each family because some newborn children (e.g. twins) were clustered within families. Early‐life factors, including maternal characteristics, newborn child characteristics, neonatal inflammatory proteins, and neonatal comorbidities, were all entered into the models simultaneously first and then adjusted in a stepwise fashion to reach a final model. Based on the social disadvantage index (defined earlier), we created a binary variable defining two risk groups (i.e. those with a social disadvantage composite of 0 vs those with a social disadvantage composite of 1 or more) to investigate our three targeted interactions: (1) social disadvantage risk group × ventilation use; (2) social disadvantage risk group × bacteremia; and (3) social disadvantage risk group × WMI. Finally, to examine how lower cognitive functioning at age 2 years (i.e. BSID‐II MDI less than 1SD below the mean) influenced the progression of cognitive scores over time, the linear mixed effect model was repeated for each outcome at ages 10 years and 15 years, covarying for the presence of a low BSID‐II MDI score.

The significance level was set at 0.05; all tests were two‐sided unless otherwise specified. All analyses were performed using R v4.1.1 (R Foundation for Statistical Computing, Vienna, Austria).

### Management of missing data

There were no specific trends in missing values for the three cognitive outcomes over time. The missing data rates for non‐verbal cognition were 7.7%, 1.2%, and 8.0% at ages 2 years, 10 years, and 15 years respectively. The missing data rates for verbal abilities were 7.7%, 1.1%, and 7.1% at ages 2 years, 10 years, and 15 years respectively. Thus, we assumed they were missing at random in the main regression analysis and that followed a likelihood‐based approach based on all available data.

## RESULTS

### Sample characteristics

The final sample included 659 participants who had at least two cognitive assessments at 2 years, 10 years, and 15 years of age. When participants who were included in the longitudinal sample were compared to those not included (*n* = 539), several group differences were noted; the tested group was less diverse (i.e. more White and non‐Hispanic), had less social disadvantage, but did not differ from participants aged 15 years with regard to neonatal characteristics.

As shown in Table 1, about two‐thirds of mothers were between 21 years and 35 years of age, about two‐thirds identified as White, and about 10% identified as Hispanic. In terms of risk factors, about 37% had a high school education or lower; 32% lacked private health insurance; 20% received supplemental nutrition assistance; and 19% were unmarried. On the social disadvantage index, approximately 40% reported one or more social risks.

**Table 1:** Description of the ELGAN sample at study entry (*n* = 659).

Maternal characteristic	*n* (%)
Age (years)^a^	
< 21	79 (12.1)
21–35	431 (66.2)
> 35	141 (21.7)
Ethnicity	
White	430 (66.1)
Black	154 (23.7)
Not specified	60 (9.2)
Hispanic	
Yes	56 (8.6)
No	592 (90.9)
Education (years)	
≤ 12 (high school)	235 (36.1)
> 12 to < 16	143 (22.0)
≥ 16 (college or higher)	255 (39.2)
Private health insurance	
Yes	438 (67.3)
No	203 (31.2)
Supplemental nutritional assistance	
Yes	132 (20.3)
No	517 (79.7)
Married/partnered	
Yes	525 (80.6)
No	125 (19.2)
Social disadvantage composite^b^	
0	298 (49.0)
1	126 (20.7)
2	90 (14.8)
3–4	94 (15.5)
*Newborn child characteristic*	
Sex	
Male	330 (50.7)
Female	321 (49.3)
Gestational age (full weeks)	
23–24	137 (21.0)
25–26	298 (45.8)
27	216 (33.2)
Birthweight (g)	
≤ 750	244 (37.5)
751–1000	278 (42.7)
> 1000	129 (19.8)
Weight for gestational age *z*‐score	
< −2	41 (6.3)
≥ − 2 to < −1	83 (12.7)
≥ − 1	527 (81.0)
Multiple birth	
Yes	197 (30.3)
No	454 (69.7)
*Neonatal inflammation*	
Sustained elevations of inflammatory proteins	
0–1	492 (75.6)
2–3	106 (16.3)
4+	53 (8.1)
*Neonatal comorbidity*	
White matter injury	
Yes	92 (14.0)
Bacteremia/sepsis	
Definite	247 (37.9)
Suspected	219 (33.6)
Not present	185 (28.4)
Bronchopulmonary dysplasia	
None	303 (46.5)
Oxygen only	288 (44.2)
Ventilation	60 (9.2)

^a^ Maternal age was not recorded for eight individuals.

^b^ Maternal education, health insurance status, receipt of supplemental nutrition assistance, and marital/partnered status at child's birth.

For the children (Table 1), the sample was divided relatively equally according to sex, with about 38% from multiple births. About 21% were born between 23 weeks and 24 weeks, 46% between 25 weeks and 26 weeks, and 33% at 27 weeks. About a quarter of the sample had two or more sustained inflammatory proteins present in their first 2 weeks of postnatal life. With respect to neonatal comorbidities, percentages ranged widely from about 14% for children with WMI to 53% for children with BPD to 72% with either suspected or definite bacteremia..

### Verbal abilities

As seen in Table 2, after adjustment of other covariates, the sample showed a small but significant positive change in verbal abilities over 13 years (*β* = 0.08, 95% confidence interval [CI] = −0.59 to 0.01, *p* < 0.001). This small, but positive trajectory was not uniform, with significant variability in individual case trajectories over time. Furthermore, our multivariable regression analysis showed that male sex (*p* < 0.001), 23 weeks to 24 weeks gestational age compared to 27 weeks (*p* = 0.005), mild‐to‐moderate BPD (*p* = 0.004), severe BPD (*p* < 0.001), and an inflammation index of 4 or more (indicating 4 or more elevations of inflammatory proteins in the first 2 weeks of life; *p* = 0.007) were associated with significant declines in verbal scores over time after controlling for other variables in the model. A gestational age of 25 weeks to 26 weeks, birthweight *z*‐score, bacteremia, and an inflammation index of 1 to 3 were not associated with a change in cognitive scores.

**Table 2:** Linear mixed model showing the main effects and interactions for verbal abilities (*n* = 1737 visits).

Predictor	Parameter estimate (SE)	95% CI	*p*
Intercept	−0.30 (0.15)	−0.59 to −0.01	0.05
Age	0.08 (0.004)	0.07 to 0.09	< 0.001
Social disadvantage composite	−0.30 (0.07)	−0.44 to −0.16	< 0.001
Sex (male)	−0.44 (0.08)	−0.60 to −0.28	< 0.001
Gestational age 23–24 weeks^a^	−0.37 (0.13)	−0.63 to −0.12	0.005
Gestational age 25–26 weeks^a^	−0.16 (0.11)	−0.38 to 0.06	0.14
Gestational weight for age *z*‐score	−0.07 (0.04)	−0.15 to 0.09	0.11
WMI (yes)	−1.27 (0.22)	−1.70 to −0.84	< 0.001
Bronchopulmonary dysplasia oxygen only^b^	−0.28 (0.09)	−0.46 to −0.10	0.004
Bronchopulmonary dysplasia ventilation^b^	−0.71 (0.18)	−1.06 to −0.36	< 0.001
Bacteremia (sepsis) definite^c^	0.20 (0.14)	−0.07 to 0.47	0.14
Bacteremia (sepsis) suspected^c^	0.29 (0.14)	0.02 to 0.56	0.06
2–3 elevations of inflammatory proteins^d^	−0.14 (0.12)	−0.38 to 0.10	0.24
4 or more elevations of inflammatory proteins^d^	−0.41 (0.15)	−0.70 to −0.12	0.007
Social risk score X WMI (yes)	0.35 (0.13)	0.10 to 0.61	0.005

*Note*: Interactions for the social disadvantage composite group × ventilation (*p* = 0.49) and social disadvantage composite × bacteremia (*p* = 0.47) were not significant and were removed from the model.

^a^ Reference groups are those with a gestational age of 27 weeks.

^b^ Reference groups are those not requiring either supplemental oxygen or assisted ventilation.

^c^ Reference groups are those with neither suspected nor definite sepsis.

^d^ Reference groups are those with 0 or 1 elevation of inflammation‐related proteins.

Abbreviations: CI, confidence interval; SE, standard error; WMI, white matter injury.

While the main effects of the social disadvantage index (*p* < 0.001) and WMI (*p* = 0.001) were significant, suggesting that more social disadvantage and the presence of early WMI were associated with fewer gains in cognitive abilities; the interaction between the social disadvantage index groups and WMI was also significant (*β* = 0.35, 95% CI = 0.10–0.61, *p* = 0.005). While those with no social disadvantage showed higher scores at each time point than those with any social disadvantage risk, the presence of WMI moderated the impact of social disadvantage risk; specifically, a difference in scores across social disadvantage index groups was not found among children with perinatal WMI. The interactions for the social disadvantage index groups according to ventilation and bacteremia were not significant.

When controlling for other variables, and then including lower BSID‐II MDI, verbal scores declined over time at the 10‐year and 15‐year time points. Children with a lower BSID‐II MDI at age 2 years showed nearly a 1SD decline in verbal abilities over time (*β* = −0.89, standard error [SE] = 0.10, 95% CI = −1.09 to −0.69, *p* < 0.001).

### Non‐verbal abilities

Non‐verbal abilities also showed a small positive change over time (*β* = 0.05, 95% CI = 0.04–0.06, *p* < 0.001) after adjusting for all covariates. However, as shown in Table 3, male sex (*p* < 0.001), 23 weeks to 24 weeks gestational age compared to 27 weeks (*p* < 0.001), presence of WMI (*p* < 0.001), mild‐to‐moderate BPD (*p* = 0.01), severe BPD (*p* < 0.001), and an inflammation index of 4 or more (*p* = 0.002), predicted a slower rate of gain in non‐verbal abilities. All of these findings were small to large in magnitude. No other variables were associated with change in non‐verbal abilities over time.

**Table 3:** Linear mixed model showing the main effects for non‐verbal abilities (*n* = 1725 visits).

Predictor	Parameter estimate (SE)	95% CI	*p*
Intercept	−0.28 (0.14)	−0.55 to −0.01	0.05
Age	0.05 (0.004)	0.04 to 0.06	< 0.001
Social disadvantage composite	−0.26 (0.06)	−0.38 to −0.14	< 0.001
Sex (male)	−0.29 (0.08)	−0.45 to −0.13	< 0.001
Gestational age 23–24 weeks^a^	−0.48 (0.12)	−0.72 to −0.25	< 0.001
Gestational age 25–26 weeks^a^	−0.18 (0.10)	−0.38 to 0.02	0.07
Gestational weight for age *z*‐score	0.07 (0.04)	−0.01 to 0.15	0.09
WMI (yes)	−1.24 (0.22)	−1.67 to −0.81	< 0.001
Bronchopulmonary dysplasia oxygen only^b^	−0.22 (0.09)	−0.40 to −0.04	0.01
Bronchopulmonary dysplasia ventilation^b^	−0.75 (0.18)	−1.10 to −0.40	< 0.001
Bacteremia (sepsis) definite^c^	0.15 (0.13)	−0.11 to 0.41	0.25
Bacteremia (sepsis) suspected^c^	0.26 (0.14)	−0.01 to 0.53	0.06
2–3 elevations of inflammatory proteins^d^	−0.10 (0.11)	−0.32 to 0.12	0.34
4 or more elevations of inflammatory proteins^d^	−0.44 (0.14)	−0.71 to −0.17	0.002
Social disadvantage composite group X WMI (yes)	0.33 (0.12)	0.10 to 0.57	0.007

*Note*: Interactions for social disadvantage composite group × ventilation (*p* = 0.78) and social disadvantage composite × bacteremia (*p* = 0.60) were not significant and were removed from the model.

^a^ Reference groups are those with a gestational age of 27 weeks.

^b^ Reference groups are those not requiring either supplemental oxygen or assisted ventilation.

^c^ Reference groups are those with neither suspected nor definite sepsis.

^d^ Reference groups are those with 0 or 1 elevation of inflammation‐related proteins.

Abbreviations: CI, confidence interval; SE, standard error; WMI, white matter injury.

The main effects for the social disadvantage index (*p* < 0.001) and WMI (*p* = 0.001) were significant, with a large effect size for the presence of early WMI; however, the interaction between social disadvantage index group and perinatal WMI was also significant (*β* = 0.33, 95% CI = 0.10–0.57, *p* = 0.007). Like the trajectory for verbal abilities, those with no social disadvantage showed higher scores at each time point than those with any social disadvantage; however, the presence of early WMI moderated the positive impact of no social disadvantage. The interactions for the social disadvantage index groups according to ventilation, and the social disadvantage index groups according to bacteremia were not significant.

Children with lower MDI scores at age 2 years did not exhibit any major shifts in their non‐verbal scores at ages 10 years and 15 years (*β* = −0.09, SE = 0.14, 95% CI = −0.36 to 0.18, *p* = 0.54).

### Combined cognitive abilities

Results from the linear mixed effect model showed a small but significant increase in overall cognitive scores with age across the three time points from infancy into middle adolescence (*p* < 0.001). As seen in Table 4, after adjustment for the covariates, male children (*p* < 0.001), 23 weeks to 24 weeks gestational age (*p* < 0.001), mild‐to‐moderate BPD (*p* = 0.005), severe BPD (*p* < 0.001), and an inflammation index of 4 or more (*p* = 0.002) showed a slower change in scores over time. In particular, having a gestational age of 23 weeks to 24 weeks, compared to 27 weeks, and severe BPD had moderate‐to‐strong effect sizes with respect to change in overall cognitive abilities. Higher gestational age, gestational weight *z*‐score, bacteremia, and inflammation indices less than 4 were not significant contributors to change in cognitive scores over time.

**Table 4:** Linear mixed model showing the main effects and interactions for combined abilities (*n* = 1769 visits).

Predictor	Parameter estimate (SE)	95% CI	*p*
Intercept	−0.24 (0.15)	−0.53 to 0.05	0.10
Age	0.06 (0.004)	0.05 to 0.07	< 0.001
Social disadvantage composite	−0.30 (0.06)	−0.42 to −0.18	< 0.001
Sex (male)	−0.38 (0.08)	−0.54 to −0.22	< 0.001
Gestational age 23–24 weeks^a^	−0.45 (0.13)	−0.71 to −0.20	< 0.001
Gestational age 25–26 weeks^a^	−0.18 (0.10)	−0.38 to 0.02	0.08
Gestational weight for age *z*‐score	0.07 (0.04)	−0.01 to 0.15	0.10
WMI (yes)	−1.26 (0.22)	−1.69 to −0.83	< 0.001
Bronchopulmonary dysplasia oxygen only^b^	−0.26 (0.09)	−0.44 to −0.08	0.005
Bronchopulmonary dysplasia ventilation^b^	−0.75 (0.18)	−1.10 to −0.40	< 0.001
Bacteremia (sepsis) definite^c^	0.16 (0.13)	−0.10 to 0.42	0.23
Bacteremia (sepsis) suspected^c^	0.26 (0.14)	−0.01 to 0.53	0.06
2–3 elevations of inflammatory proteins^d^	−0.13 (0.11)	−0.35 to 0.09	0.24
4 or more elevations of inflammatory proteins^d^	−0.45 (0.15)	−0.74 to −0.16	0.002
Social disadvantage composite group X WMI (yes)	0.34 (0.12)	0.11 to 0.58	0.006

*Note*: Interactions for social disadvantage composite group × ventilation (*p* = 0.54) and social disadvantage composite × bacteremia (*p* = 0.54) were not significant and were removed from the model.

^a^ Reference groups are those with a gestational age of 27 weeks.

^b^ Reference groups are those not requiring either supplemental oxygen or assisted ventilation.

^c^ Reference groups are those with neither suspected nor definite sepsis.

^d^ Reference groups are those with 0 or 1 elevation of inflammation‐related proteins.

Abbreviations: CI, confidence interval; SE, standard error; WMI, white matter injury.

While the main effects for the social disadvantage index (*p* < 0.001) and WMI (*p* < 0.001) were significant, with a large effect size for the early presence of WMI, the interaction of the social disadvantage index groups with WMI also was significant (*β* = 0.34, 95% CI = 0.11–0.58, *p* = 0.006). As with the verbal and non‐verbal cognitive trajectories, this interaction showed that when perinatal WMI was present, children with and without social disadvantage had similar combined cognitive scores. This interaction is illustrated in Figure 1. Additionally, as shown in Figure 1, children in the group with early WMI with social risk continued to make positive cognitive gains over the three time points. The interactions for the social disadvantage index according to ventilation use and social risk group according to bacteremia were not significant.

**Figure 1:**
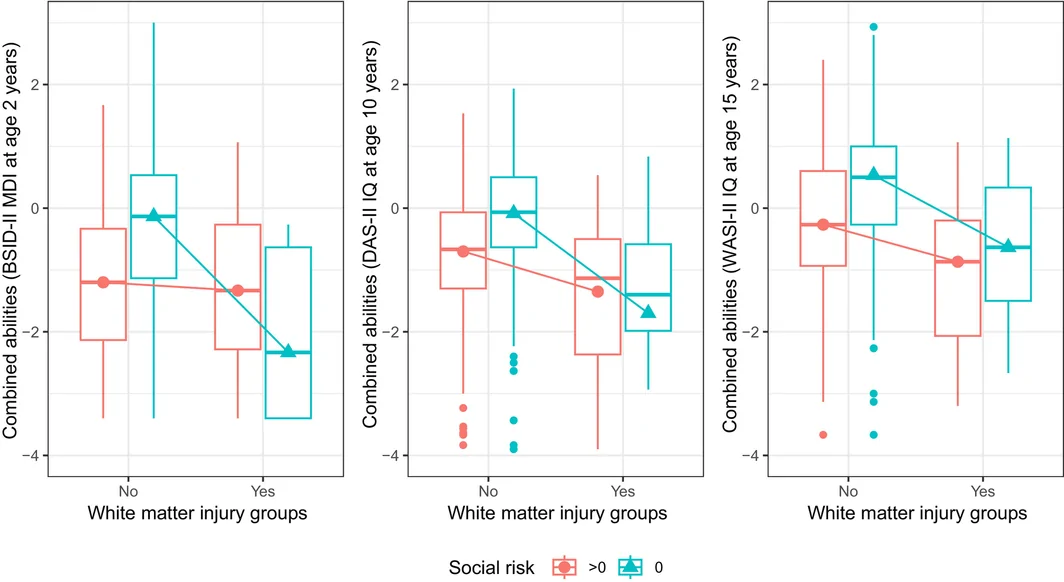
Social disadvantage index group X WMI interaction box plots showing the 25th and 95th centiles for combined abilities at ages 2 years, 10 years, and 15 years. Abbreviations: BSID‐II, Bayley Scales of Infant Development, Second Edition; DAS‐II, Differential Ability Scales, Second Edition; WASI‐II, Wechsler Abbreviated Scale of Intelligence, Second Edition; WMI, white matter injury.

When controlling for the other covariates, lower MDI at age 2 years was associated with nearly a 1SD decline in scores between the ages of 10 years and 15 years (*β* = −0.86, SE = 0.09, 95% CI = −1.04 to −0.68, *p* < 0.001).

## DISCUSSION

Consistent with the previous literature,^1^, ^2^, ^3^, ^4^, ^5^, ^6^, ^7^, ^8^, ^9^ the findings from this longitudinal cohort study tracking infants born extremely preterm into middle adolescence revealed that this sample demonstrated small but significant positive gains in verbal, non‐verbal, and overall cognitive abilities; however, the magnitude of these changes was probably of little clinical significance. Predictors of decline in cognitive abilities included social disadvantage at birth, male sex, low gestational age, and a neonatal medical disorder (e.g. BPD, WMI); however, the results do not support any differential pattern of predictors between verbal and non‐verbal outcomes. A lower level of developmental attainment in infants at 2 years was associated with a significant decline in scores over time, representing nearly 1SD in magnitude in verbal and combined cognitive abilities.

Relatively new is our finding that the presence of four or more repeated elevations of inflammatory proteins in the first 2 postnatal weeks of life was associated with a decline in cognitive scores of nearly half an SD between the ages of 2 years and 15 years. Previously, we reported that such elevations were predictors of cognitive functioning at ages 2 years and 10 years.^26^ These selective inflammatory proteins have been linked to several neurological and cognitive difficulties;^27^, ^28^ ongoing studies of these early neonatal biomarkers may yield potential targets for early interventions in children born extremely preterm.

Another relatively new finding was that the effect of having no social disadvantage was evident only among children without WMI; that is, when early WMI was present, the positive impact of social advantage was lessened. The latter finding agrees with studies describing a long‐term impact of early‐life hazardous exposure on brain development^19^, ^20^, ^21^ but contrasts with the findings of Benavente‐Fernández et al.,^10^ who found no effect of early brain injury among children whose mothers had the highest levels of educational achievement. Potential explanations for the difference between our conclusions and those of Benavente‐Fernández et al. include the lower gestational age in our sample, the extension of our longitudinal follow‐up into middle adolescence, and how social disadvantage factors were operationalized. Nonetheless, it is important to note that children in the group with early WMI with social risk continued to make overall positive cognitive gains over the three time points (Figure 1).

Given the complex interplay between social factors and brain development, it is important for this interaction to receive ongoing scientific scrutiny, perhaps by examining whether neonatal morbidities are on a causal pathway to cognitive outcomes.

While the ELGAN study is one of the largest longitudinal cohorts of infants born extremely preterm assembled to date, we acknowledge several limitations. First, the study was not designed to have a control group of children born at term, so we cannot draw inferences as to how our study participants compare with peers born at term. Second, while the findings pertaining to the early inflammatory proteins are relatively new for longitudinal findings, the clinical usefulness of these protein measures is currently limited because routine measurements are not a component of standard clinical practice. Third, the use of three different measures of cognitive functioning, while standardized and age‐appropriate, may have created unknown measurement errors in our longitudinal predictions. Finally, while we examined cognitive trajectories and their early predictors in our entire sample, future studies should explore the possibility that there may be differential patterns of early predictors if specific subgroups born extremely preterm are targeted for study.

## Conclusions

This study adds to the evidence that early social and neonatal morbidity factors are important in predicting long‐term change in cognitive scores.^30^ Our findings show that children born extremely preterm and showing early medical complications, including four or more elevations in circulating inflammatory proteins at birth, or more socially disadvantaged, are at particularly high risk for a decline in cognitive scores over time. Efforts to address several social determinants of health, particularly in males and those with associated neonatal morbidities, may lessen the downstream impact on later cognitive functioning in children born extremely preterm.

## Supporting information


**Figure S1:** Participants of the ELGAN study from enrollment to the present.

## Data Availability

Data for all variables except 15‐year outcomes can be obtained from Archived Clinical Research Data Sets, National Institute of Neurological Disorders and Stroke (nih.gov). Data on 15‐year outcomes can be obtained from the public use data set available on the NICDH DASH website.
