# Supplementary material for: Early predictors of cognitive trajectories from birth to adolescence in children born extremely preterm
Source: Dev Med Child Neurol. 2026 Feb 26;68(11):1551–60. doi: 10.1111/dmcn.70211 (PMC13649299; doi:10.1111/dmcn.70211)
Supplement: Supplementary file 1 [file DMCN-68-1551-s001.pdf]

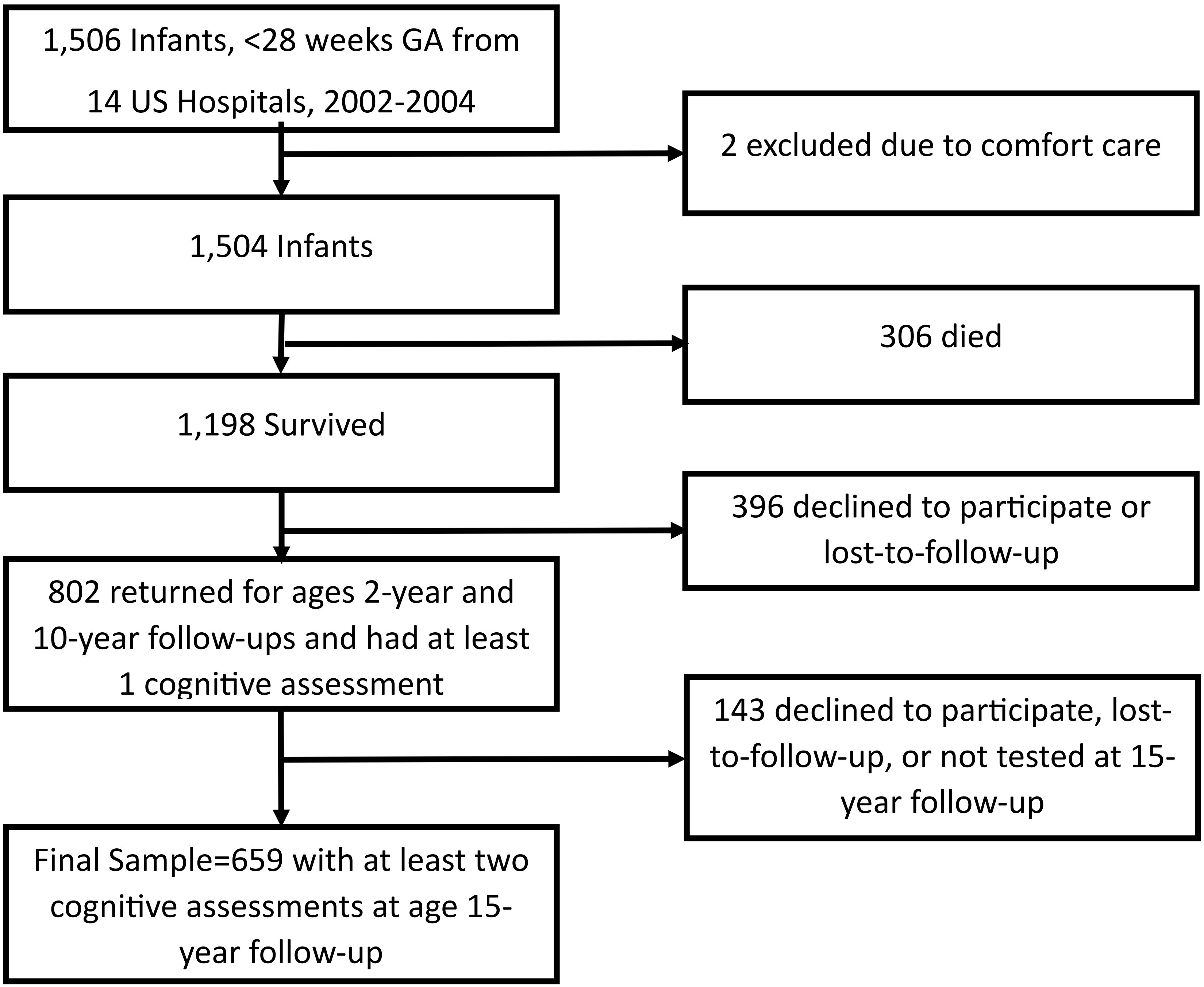
1,506 Infants, <28 weeks GA from 14 US Hospitals, 2002-2004
2 excluded due to comfort care
1,504 Infants
306 died
1,198 Survived
396 declined to participate or lost-to-follow-up
802 returned for ages 2-year and 10-year follow-ups and had at least 1 cognitive assessment
143 declined to participate, lost-to-follow-up, or not tested at 15-year follow-up
Final Sample=659 with at least two cognitive assessments at age 15-year follow-up
